# The Role of Disopyramide after Myocardial Infarction

**Published:** 1976

**Authors:** M. B. S. Jones

**Affiliations:** Consultant Physician, Gloucester Royal Hospital


### The Role of Disopyramide after Myocardial Infarction

**Dr. M. B. S. Jones,**
Consultant Physician, Gloucester Royal Hospital

Between 1974 and 1975, we conducted a double blind trial in patients with post myocardial infarction to compare oral disopyramide and placebo in the Coronary Care Units of St. Mary's Hospital, Paddington and Edgware General Hospital.

We studied 95 patients with confirmed myocardial infarction, of whom 46 received disopyramide and 49 received placebo. The groups were evenly matched in their Peel prognostic indices. The mean time of entry into the trial after infarction was 8 hours in both groups. Since we were then using a relatively untried drug, we used it in the good risk myocardial infarction patients. Therefore, all unconscious patients, patients with a systolic blood pressure of less than 80 mmHg for 2 hours, patients with heart block or with obvious risks of developing anti-cholinergic problems, and patients who initially had an arrhythmia were excluded.

Immediately after acceptance into the trial, disopyramide or placebo was administered on a blind statistically randomised basis, and a 24 hours' continuous ECG tape recording started. The tapes were analysed retrospectively on an automatic arrhythmia detection system, and checked manually.

Patients were withdrawn from the trial if they developed an arrhythmia requiring treatment within the first 6 hours, since effective serum levels would not have been reached in that time.

We classified the arrhythmias into three types:

1. Ventricular premature beats (5 per minute), R. on T., bigeminy, multifocal ectopics, runs of ventricular premature beats, ventricular tachycardia and fibrillation.
2. The supraventricular tachycardias.
3. Heart block.

Twenty-one patients in the disopyramide group and 42 in the placebo group required further anti-arrhythmic treatment ($p<0.0005$). 15 ventricular arrhythmias occurred on disopyramide, compared with 29 in patients receiving placebo ($p<0.01$).

Eight cases of ventricular premature beats (>5/min.) occurred in the disopyramide group, and 21 in the placebo group ($p<0.01$). The incidence of multifocal or R. on T. ventricular premature beats was not significantly different between the disopyramide and placebo groups. Only 4 patients on disopyramide developed ventricular tachycardia compared with only 14 on the placebo ($p<0.05$). The incidence of ventricular fibrillation or supraventricular arrhythmias was not signficant between the two groups.

None of the patients on disopyramide developed any degree of heart block, whereas 7 developed varying degrees on placebo ($p<0.05$).

There was no significant difference between the two groups in the incidence of complications observed.

Only one patient on disopyramide re-infarcted during the hospital stay, compared with 9 patients on placebo ($p<0.05$). There were two deaths in the disopyramide group compared with 5 on the placebo.

Disopyramide gave a signficant prophylactic protection against potentially dangerous ventricular arrhythmias, but little against supraventricular arrhythmias, a surprising apparent protection against the development of heart block, a possible reduction in the extension of the original infarction and no significant predominance of side-effects.

**Reference**

Jennings, G., Model, D. G., Jones, M. B. S., Turner, P. P., Besterman, E. M. M., and Kidner, P. H. (1976) Oral disopyramide in prophylaxis of arrhythmias following myocardial infarction. Lancer, 1, 51-54.

